# Arbuscular Mycorrhizal Fungi Contribute to Phosphorous Uptake and Allocation Strategies of *Solidago canadensis* in a Phosphorous-Deficient Environment

**DOI:** 10.3389/fpls.2022.831654

**Published:** 2022-03-24

**Authors:** Shanshan Qi, Jiahao Wang, Lingyun Wan, Zhicong Dai, Dalva Maria da Silva Matos, Daolin Du, Suhelen Egan, Stephen P. Bonser, Torsten Thomas, Angela T. Moles

**Affiliations:** ^1^Key Laboratory of Modern Agricultural Equipment and Technology, Ministry of Education, School of Agricultural Engineering, Jiangsu University, Zhenjiang, China; ^2^School of the Environment Safety Engineering, Jiangsu University, Zhenjiang, China; ^3^Evolution & Ecology Research Centre, School of Biological, Earth and Environmental Sciences, UNSW Sydney, Sydney, NSW, Australia; ^4^Guangxi Botanical Garden of Medicinal Plants, Nanning, China; ^5^Centre for Marine Science and Innovation, School of Biological, Earth and Environmental Sciences, UNSW Sydney, Sydney, NSW, Australia; ^6^Jiangsu Collaborative Innovation Center of Technology and Material of Water Treatment, Suzhou University of Science and Technology, Suzhou, China; ^7^Departamento de Hidrobiologia, Federal University of São Carlos, UFSCar, São Carlos, Brazil

**Keywords:** arbuscular mycorrhizal fungi, invasive clonal plant, nutrient limitation, phosphorus uptake, Canada goldenrod, sterile culture system

## Abstract

Arbuscular mycorrhizal fungi (AMF) can facilitate the uptake of limiting or inaccessible nutrients by plants. However, the importance of AMF for invasive plants under phosphorus (P) limitation is poorly well understood because of the presence of non-focal microorganisms, such as endophytes or rhizosphere bacteria. In this study, we investigated how an invasive clonal plant *Solidago canadensis* benefits from the AMF *Glomus intraradices* by using a completely sterile culturing system, which is composed of aseptic seedlings, a pure AMF strain, and a sterile growth environment. We found that the colonization rate, abundance, and spore production of AMF in the insoluble P treatment was more than twice as much as in the available P treatment. Plant above-ground growth was enhanced almost 50% by AMF in the insoluble P treatment. Importantly, AMF were able to facilitate P acquisition by the plant in insoluble P conditions, allowing plants to have lower investment into below-ground biomass and higher benefit/return for above-ground biomass. This study demonstrated the important contribution that AMF make to plants in phosphate-deficient environments eliminating interference from non-focal microorganisms. Our results also suggest that interaction with AMF could contribute to the invasiveness of clonal plant *S. canadensis* in a resource-deficient environment.

## Introduction

Phosphorus (P) is crucial for normal plant growth and development (Luo et al., 2019) and is often present in the soil in relatively large amounts but with low bioavailability due to the complexation with iron, calcium, and aluminum (Smith et al., 2011). Terrestrial plants have evolved two specialized strategies to increase the uptake of inorganic P from soils (Schachtman et al., 1998; Luo et al., 2019). The first is to directly take up soluble P *via* root epidermal cells and root hairs. This strategy often involves the alteration of root architecture to increase root-to-shoot ratios (Péret et al., 2014) as well as the production of organic acids, phosphatases, and P transporters to solubilize bio-unavailable P (Raghothama and Karthikeyan, 2005). The second strategy employs mutualistic symbionts, such as mycorrhizae and phosphorus-solubilizing bacteria, to increase the absorptive surface area of the root system (Smith et al., 2004; Amaya-Carpio et al., 2009; Priyadharsini and Muthukumar, 2017). Direct uptake of P through the roots requires a larger investment of plant resources than symbiont-driven P acquisition (Smith et al., 2003).

Approximately 75% of land plant species are colonized by and have mutualistic relationships with arbuscular mycorrhizal fungi (AMF) of the phylum Glomeromycota (Gutjahr et al., 2015). AMF contribute to the growth and health of their host plants by increasing nutrient acquisition, drought and salt tolerance, and also by increasing the biotic resistance to pathogens and herbivores (Kula et al., 2005; Yooyongwech et al., 2016; Lin et al., 2017). In return, AMF draw organic nutrients and photosynthates from plants (Smith and Smith, 2011). AMF can form a network with plant roots to increase inorganic phosphorus acquisition by producing organic acids and phosphatases (Lee et al., 2014; Majewska et al., 2017). Recent studies also revealed that AMF possess various key genes involved in the phosphate response signal transduction pathway (Salvioli et al., 2016; Venice et al., 2020). However, molecular mechanisms of phosphate transport and metabolism are still need further study (Xie et al., 2022).

Invasive plants cause both huge economic losses and severe ecological problems (Vila et al., 2011). Successful invasive plants often possess rapid growth abilities and have strong survival under adversity (Dai et al., 2016a; Chen et al., 2019). Invasive plants are also often influenced by mutualistic interactions with AMF (Majewska et al., 2015; van Kleunen et al., 2018; Chen et al., 2019), which increase their competitive abilities and facilitate invasion of new habitats (Callaway et al., 2001; Yuan et al., 2014; Zhang et al., 2017). For example, Dong et al. (2021) found that invasive plants grew larger and with lower competitive suppression with AMF colonization. However, Majewska et al. (2017) observed that the effects of AMF on the growth of two invasive plants, *Rudbeckia laciniata* and *Solidago gigantea*, were different depending on various AMF species and soil types.

Our focus in this study is on the invasive clonal plant *Solidago canadensis* L. (Asteraceae), a North American plant that has successfully invaded Europe, Asia, and Oceania (Jin et al., 2004). *S. canadensis* often forms monocultures in its invaded ranges, likely due to having both sexual and asexual clonal reproduction and allelopathic impacts on competitors (Dong et al., 2006). *S. canadensis* has become a notorious weed in various habitats in East China, including roadsides, abandoned and agricultural fields, and even open barren areas (Wan et al., 2018b; Ren et al., 2019). Wan et al. (2018b) found that, in its invaded ranges, *S. canadensis* tends to be found in areas with nutrient-poor soil, where available P is low because of the loss of organic material. *S. canadensis* is known to have strong performance even under very low P availability (Yu et al., 2016; Wan et al., 2018a). One possibility is that *S. canadensis* achieves success under low P conditions through its association with AMF, such as *G. intraradices*. Although *G. intraradices* can be found in almost all soils and is a generalist fungus that associates with many plant taxa (van der Heijden et al., 2015), there is no existing evidence that *G. intraradices* forms a mutualistic relationship with *S. canadensis*. Thus, *G. intraradices* was chosen as a representative AMF species in this work to study the effects on the growth of *S. canadensis.*

Our aim is therefore to determine how AMF affects invasive clonal plant *S. canadensis* to achieve high performance in phosphorus-deficient soils. We predicted that associations with AMF would increase the ability of plants to absorb phosphorus and allow them to change their resource allocation strategy to favor increased above-ground biomass (Berta et al., 1993; Vance et al., 2003). Our study extends previous work in this field by using axenic conditions to avoid potential confounding factors caused by the presence of non-focal microorganisms, such as endophytes or rhizosphere bacteria, that are known to affect plant growth and nutrient uptake (Chen et al., 2006; Rout et al., 2013; Afkhami and Strauss, 2016; Dai et al., 2016b; McLeod et al., 2016; Priyadharsini and Muthukumar, 2017). We used pure cultures of the AMF *G. intraradices* with aseptic seedlings of *S. canadensis* grown under completely sterile culture conditions to determine:

(1) Does *G. intraradices* form a mutualistic relationship with *S. canadensis*?
(2) Does the relationship between *G. intraradices* and *S. canadensis* vary with nutrient availability?
(3) Do P availability and colonization by *G. intraradices* affect the growth of *S. canadensis*?
(4) Does *G. intraradices* increase phosphate uptake of *S. canadensis*?
(5) What effect does *G. intraradices* have on the resource allocation strategy of *S. canadensis*?

## Materials and Methods

We began by asking whether the AMF *G. intraradices* forms a mutualistic relationship with *S. canadensis* seedlings by growing aseptic seedlings with a monoxenic culture of AMF. Aseptic seedlings (Supplementary Figure 1) were produced from fresh shoots of *S. canadensis* according to the method by Dai et al. (2016b). Briefly, fresh apical buds of *S. canadensis* were surface-sterilized with 75% ethanol for 1 min and 5% sodium hypochlorite solution for 10 min, and then washed five times with sterilized distilled water. These apical buds were then put into sterilized Murashige and Skoog (MS) solid medium supplemented with 0.8 mg⋅L^–1^ 6-benzylaminopurine, 0.1 mg⋅L^–1^ 1-naphthaleneacetic acid, and 0.8 mg⋅L^–1^ silver nitrate. After clusters of axillary buds proliferated (∼50 days of culturing), the aseptic shoots were cut and maintained in MS medium for about 5 days to obtain seedlings with roots for further treatments. The absence of contaminant microorganisms in the seedlings (Supplementary Figures 2, 4) was assessed using both the coating plate method and 16S/18S rRNA gene amplification (Dai et al., 2016b).

**FIGURE 1:**
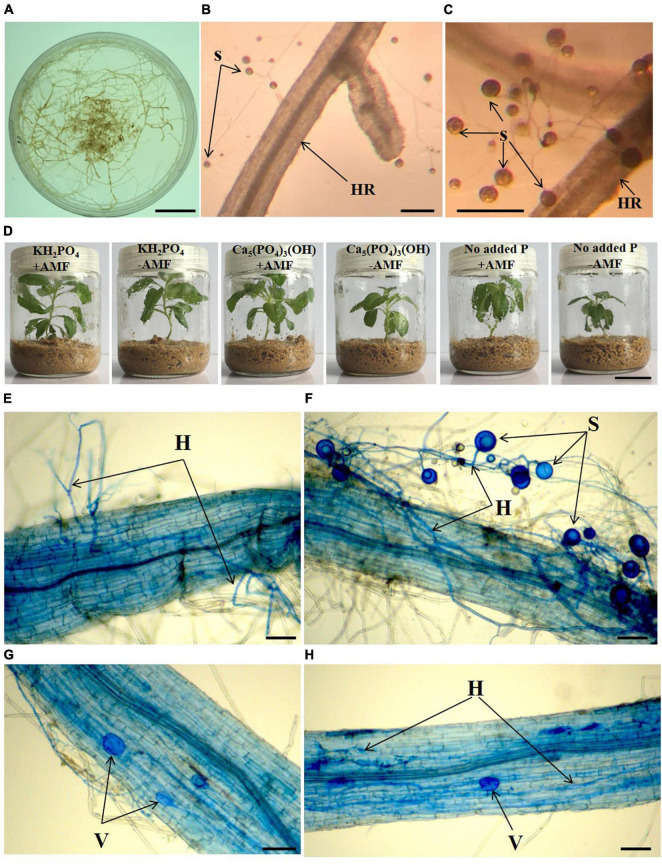
Monoxenic culture system of *Glomus intraradices* **(A–C)**, aseptic growth conditions **(D)** of *Solidago canadensis* grown in different phosphorus treatments with or without arbuscular mycorrhizal fungi (AMF) colonization, and mycorrhizal root colonization of *S. canadensis* in aseptic seedling culture system **(E–H)**. HR, hairy root, H, hyphae, S, spore, V, vesicles, +AMF- with AMF colonization, -AMF- without AMF colonization. Bars in **(A,D)** = 2 cm, bars in **(B,C)** = 100 μm, bars in **(E–H)** = 25 μm.

To generate a monoxenic AMF culture, we used carrot (*Daucus carota* L.) roots transformed with the T-DNA from a tumor-inducing plasmid as the host for the pure AMF strain *G. intraradices* (Figures 1A–C). The pure AMF strain and aseptic hair-root system (available from the Key Laboratory of Ion Beam Bioengineering, Institute of Plasma Physics, Chinese Academy of Sciences) was used to obtain aseptic spores. Spores of *G. intraradices* were isolated and applied to the aseptic plant seedlings. The colonized roots from the Minimal Medium (Bécard and Fortin, 2010) which contains spores, were placed in a sterile flask. A 10-mM sodium citrate (10 times volume of medium) solution was added, and then the mixture was stirred for 10 min to separate spores from hyphae. Spores were harvested from the hairy roots of *D. carota via* initial filtration using sterile gauze, and subsequently sterile spores were collected through 0.45-μm syringe filters. Spores were washed three times using sterile water to remove the remaining sodium citrate before being suspended in sterile water.

To provide a sterile growth environment, we used glass tissue culture flasks (Dai et al., 2016b) for the different types of P supplementation to *S. canadensis* seedlings in April 2017 (Figure 1D). Washed sand (90 g) was put into each flask, sterilized at 121°C for 2 h, and then cooled to room temperature. For each flask, 25 mL modified 0.5 × Hoagland (Dai et al., 2016a) without P was added to the sand. A sterile *S. canadensis* seedling was transferred into each flask. Seedlings were grown in an incubator at 28°C and light for 16 h a day at 360 μmol⋅m^–2^⋅s^–1^ and 8 h darkness. For AMF inoculation, 1 mL *G. intraradices* spores (+ AMF) suspension (approximately 770 ± 84 spores) was added into the flasks. No spore was added as the control treatment (−AMF).

To address question 1, we measured the hyphal colonization rate and spore production in sand media in inoculated vs. non-inoculated treatments after 45 days. Plants and sand media were harvested from the flask. Roots were pulled out and the sand was shaken off and collected to count the number of spores. Roots of *S. canadensis* were sampled for hyphal staining of AMF to determine root colonization. There were five replicates for each treatment. Hyphal staining was assessed following the procedure described in Phillips and Hayman (1970). Briefly, root samples for each treatment were gently washed with distilled sterile water and cut into 2-cm pieces before being externally cleaned in 10% KOH and then acidified with 1% HCl. The surface of the root samples was then stained with 0.05% trypan blue in lactophenol before the microscopic observation of mycorrhizal colonization rate and the abundance of arbuscules in the roots (Yang et al., 2014). The abundance of colonization was classified into four classes: 0–25% colonized, 25–50% colonized, 50–75% colonized, and 75–100% colonized. No colonization or spores of *G. intraradices* were detected in the non-inoculated seedlings, confirming the lack of contamination in our experiments.

To determine whether the mutualistic relationship varied with nutrient availability (question 2), we grew *S. canadensis* seedlings in media as above, but with three different nutrient levels. Sterile conditions were established as above, and 30 mg⋅kg^–1^ P (approximately P content in Hoagland nutrient solution) was added either as soluble KH_2_PO_4_ (hereafter referred to as “Available P”) or insoluble Ca_5_(PO_4_)_3_(OH) (hereafter referred to as “Insoluble P”) to a flask and mixed evenly. No P was added to the controls (hereafter referred to as “No P addition”). Five replicates of each treatment were set up for a total of 30 flasks.

To quantify *S. canadensis*’ performance in P-deficient soil and to determine whether AMF could contribute to the growth of *S. canadensis* (question 3), treatments of available and unavailable P were set up as above. We measured a suite of traits associated with growth and resource allocation: leaf number, maximum leaf area, maximum leaf dry mass, shoot length, shoot dry mass (i.e., aboveground dry mass), root length, root dry mass, root dry mass/shoot dry mass, and specific leaf area (SLA, the ratio of maximum leaf area/dry mass). These traits were measured and calculated following Dai et al. (2016b).

To quantify the impact of AMF on phosphate uptake of *S. canadensis* (question 4), root phosphatase activity was determined following Zalamea et al. (2016). Briefly, roots were placed in a glass vial with 25 mL of 0.2 M sodium acetate–acetic acid buffer (pH 5.0) and shaken in a water bath at 28°C. The assay was initiated by adding 2.5 mL substrate (50 mM *para*-nitrophenyl phosphate, *p*NPP) and incubated for 30 min. The reaction was terminated by removing 0.5 mL of buffer solution and adding it to 4.5 mL of terminator solution (0.11 M NaOH) in a glass test tube. After vortexing, the absorption was measured at 405 nm against *para*-nitrophenol (*p*NP). Phosphatase activity was expressed in mmol *p*NP g^–1^ h^–1^ produced from *p*NPP through hydrolysis by phosphatase.

All seedlings were ground and analyzed for total P content (Fujita et al., 2010). After a digestion procedure (Kjeldahl digestion method; 1 h at 200°C and 2 h at 340°C in a mixture of concentrated sulfuric acid and 30% hydrogen peroxide), the seedlings were cooled and diluted with deionized water to 45 ml. The P content was determined colorimetrically using a UV-1200 spectrophotometer (MAPADA, Shanghai, China) following Wan et al. (2018a).

To address the effects of AMF on the resource allocation strategy of *S. canadensis* (question 5), the effective resource allocation (ERA) was calculated using the following equation (Grace et al., 2009):


(1) $$\text{ERA}\left(\%\right)=\frac{V_{+A\,M\,F}-V_{-A\,M\,F}}{V_{-A\,M\,F}}$$

where V_+*AMF*_ and V_–*AMF*_ is the value of phenotypic and physiological indicators (i.e., above- and below-ground biomass, phosphatase activity, or phosphorus content) with and without AMF inoculation, respectively.

Duncan’s multiple range tests were conducted to find if there were significant differences in colonization rate and spore number among treatments. We quantified the effects of different phosphorus treatments on the growth of *S. canadensis* with or without AMF colonization using the two-way analysis of variance (ANOVA). Duncan’s multiple range tests were also performed to determine the growth of *S. canadensis* with different treatments and also used to compare the differences of the effect of AMF on plant resource allocation. All statistical analyses were performed with the SAS statistical software 9.1, and figures were drawn with SigmaPlot 12.0 software.

## Results

**FIGURE 2:**
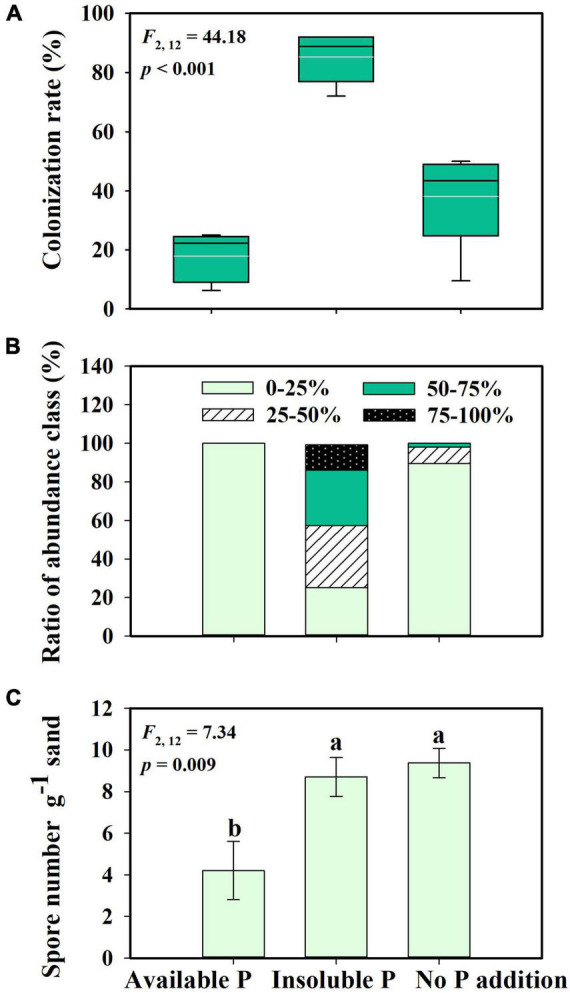
Mycorrhizal colonization rate **(A)**, hyphae abundance class **(B)**, and spore number in sand **(C)** of *Glomus intraradices* interacting with *Solidago canadensis* under different phosphorus (P) treatments. The white lines are means, the black lines are medians, the boxes show 25 to 75% quantiles in panel **(A)**. Available P, P was added as soluble KH_2_PO_4_; Insoluble P, P was added as Ca_5_(PO_4_)_3_(OH); No P addition, no P was added. Error bars are the S.E. (*n* = 5). Different letters indicate a significant difference at *p* < 0.05.

*Glomus intraradices* did form a mutualistic interaction with *S. canadensis* (Figures 1E–H). Consistent with our second hypothesis, the proportion of roots colonized by *G. intraradices* varied with P availability (*F* = 44.18; *p* < 0.001). More than 80% of roots in inoculated treatments were colonized in the Insoluble P treatment, which was significantly higher than the colonization in either the No P addition (38% colonized) or the Available P treatments (17% colonized; Figure 2A; *p* < 0.001). The abundance of *G. intraradices* on colonized roots was also higher in the Insoluble P treatment than in the Available P or No P addition treatments (Figure 2B). All roots in the Available P treatment and 90% of the roots in the No P addition treatment had below 25% colonization by *G. intraradices*, while 75% of the roots in the Insoluble P treatment were more than 25% colonized. Finally, spore numbers in the sand beneath the No P addition and Insoluble P treatments were more than twice as high as in sand from the Available P treatment (*F* = 7.34; *p* = 0.009; Figure 2C).

**FIGURE 3:**
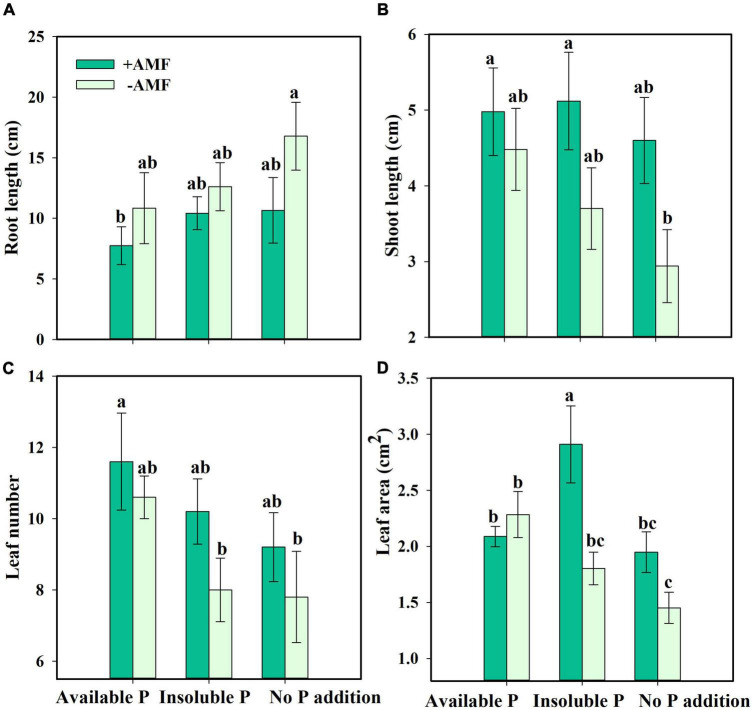
Root length **(A)**, shoot length **(B)**, leaf number **(C)**, and leaf area **(D)** of *Solidago canadensis* in different phosphorus (P) treatments. Available P, P was added as KH_2_PO_4_; Insoluble P, P was added as Ca_5_(PO_4_)_3_(OH); No P addition, no P was added. +AMF, with arbuscular mycorrhizal fungi (AMF) colonization; -AMF, without AMF colonization. Error bars are the S.E. (*n* = 5). Different letters indicate a significant difference at *p* < 0.05.

**FIGURE 4:**
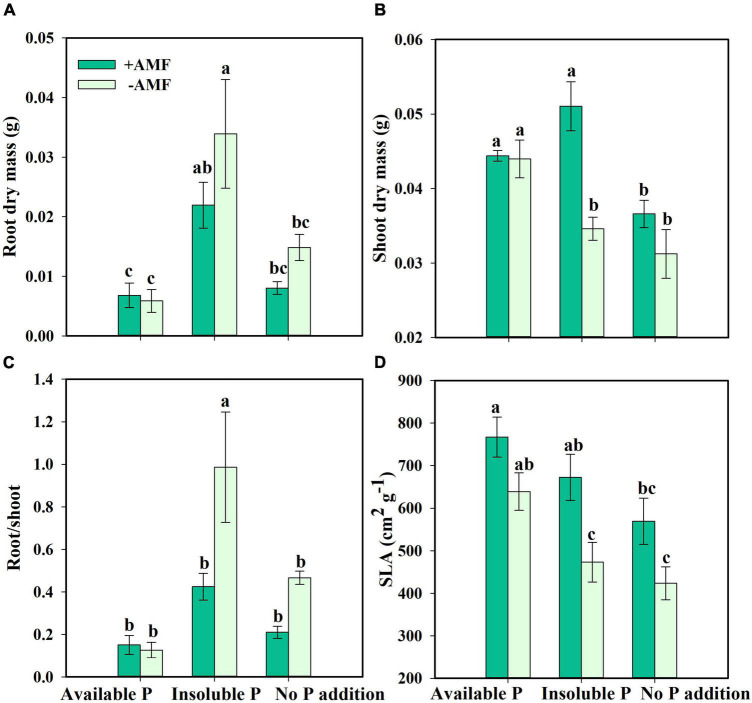
Root dry mass **(A)**, shoot dry mass **(B)**, root/shoot **(C)**, and specific leaf area [SLA, **(D)**] of *Solidago canadensis* in different phosphorus (P) treatments. Available P, P was added as KH_2_PO_4_; Insoluble P, P was added as Ca_5_(PO_4_)_3_(OH); No P addition, no P was added. +AMF, with arbuscular mycorrhizal fungi (AMF) colonization; -AMF, without AMF colonization. Error bars are the S.E. (*n* = 5). Different letters indicate a significant difference at *p* < 0.05.

Next, we asked how nutrient treatment and AMF inoculation affected the growth and functional traits of *S. canadensis* (question 3). We found that roots tended to be longer in the treatment with No P addition or AMF inoculation than in the treatment with available P and with AMF inoculation (Figure 3A). In contrast, shoot length, leaf number, and leaf area tended to increase with AMF inoculation (Figures 3B–D). Some of these effects were substantial. For example, leaf area in the Insoluble P treatment was 61% higher with AMF inoculation than in the non-inoculated treatment (1.8 vs. 2.9 cm^2^; Figure 3D and Table 1). In the Insoluble P treatment, the total dry mass tended to be greater than in the other two P treatments (Supplementary Figure 5). Compared to the non-inoculated treatment, AMF inoculation decreased both root dry mass and root to shoot ratio of plants grown in the insoluble P condition, but did not affect these traits of plants grown in the available P and no P conditions (Figures 4A,C and Table 1). On the other hand, compared to the non-inoculated treatment, the AMF inoculation increased both shoot dry mass and specific leaf area of plants grown in the insoluble P condition, but imposed no effect on these traits of plants grown in the available P or no P conditions (Figures 4B,D and Table 1). In summary, plants grown in soil with low P availability tended to shift resources from below-ground to above-ground tissues in the presence of AMF.

**TABLE 1:** Two-way ANOVAs of the effects of different phosphorus (P) treatments on the growth and functional traits of *Solidago canadensis* with/without arbuscular mycorrhizal fungi (AMF).

Growth traits	Source	*d.f.*	*F*	*p-value*
Root length	AMF	1	4.07	*0.055*
	P	2	1.84	0.181
	AMF × P	2	0.39	0.678
Shoot length	AMF	1	6.79	**0.016**
	P	2	1.52	0.239
	AMF × P	2	0.60	0.559
Leaf number	AMF	1	3.29	*0.082*
	P	2	3.45	**0.048**
	AMF × P	2	0.17	0.841
Leaf area	AMF	1	8.20	**0.009**
	P	2	5.77	**0.009**
	AMF × P	2	5.28	**0.013**
Root dry mass	AMF	1	2.85	0.104
	P	2	13.68	**0.001**
	AMF × P	2	1.13	0.339
Shoot dry mass	AMF	1	14.42	**0.001**
	P	2	10.90	**0.001**
	AMF × P	2	5.90	**0.008**
Root to shoot ratio	AMF	1	8.13	**0.009**
	P	2	13.00	**0.001**
	AMF × P	2	3.39	**0.050**
Specific leaf area	AMF	1	16.31	**0.001**
	P	2	9.55	**0.001**
	AMF × P	2	0.30	0.746

*Values of p < 0.05 are in bold. Values are in italics where 0.05 < p < 0.1.*

**FIGURE 5:**
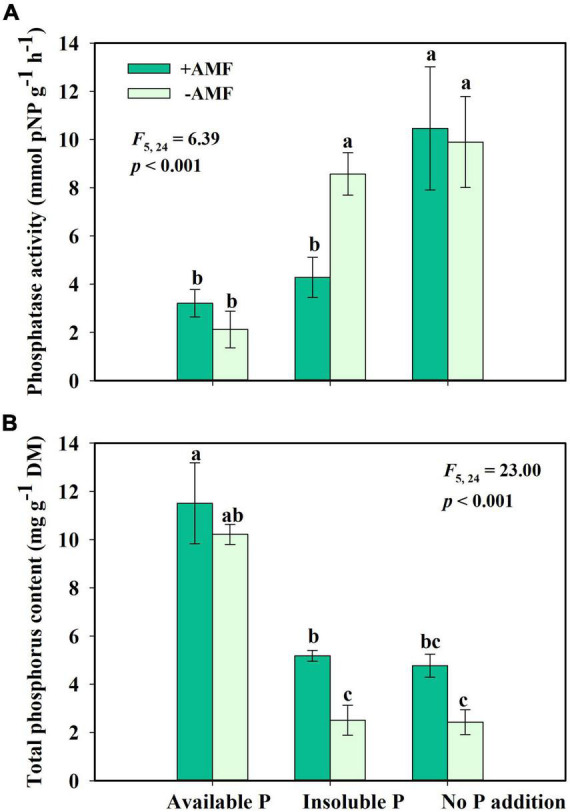
Phosphatase activity **(A)** and phosphorus content **(B)** of *Solidago canadensis* grown on three phosphorus (P) treatments with or without arbuscular mycorrhizal fungi (AMF) colonization. Available P, P was added as KH_2_PO_4_; Insoluble P, P was added as Ca_5_(PO_4_)_3_(OH); No P addition, no P was added. +AMF, with AMF colonization; -AMF, without AMF colonization. Error bars are the S.E. (*n* = 5). Different letters indicate a significant difference at *p* < 0.05.

For the fourth question, we found that AMF inoculation changed phosphatase activity and phosphate uptake of *S. canadensis*. AMF inoculation significantly decreased phosphatase activity in the Insoluble P treatment but did not affect the phosphatase activity in the Available P or No P addition treatments (Figure 5A). In the Insoluble P treatment, the AMF inoculation increased P concentration by 107% compared to the non-inoculated treatment (Figure 5B). That is, *G. intraradices* significantly promoted phosphate uptake and decreased phosphatase activity of *S. canadensis* in conditions of insoluble P (Figure 5).

**FIGURE 6:**
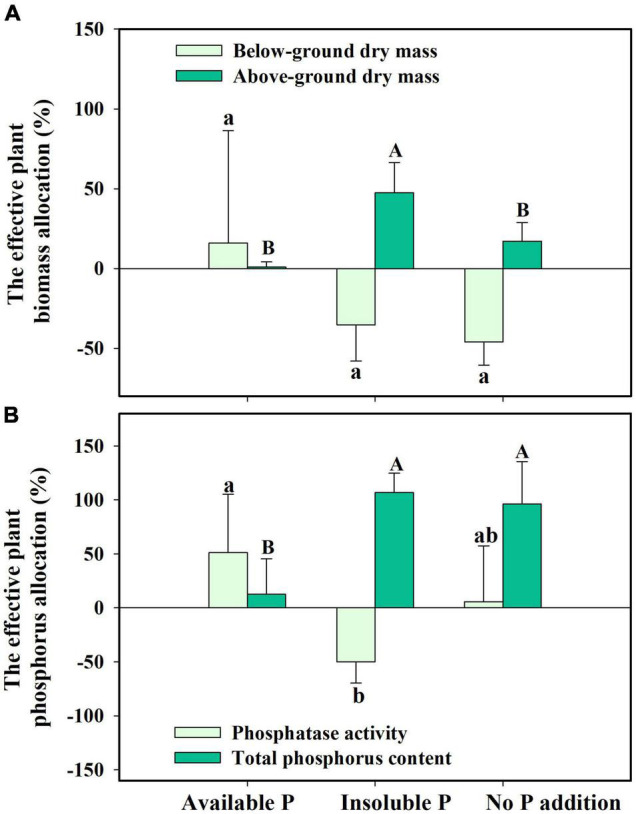
The effective resource allocation (ERA) on plant biomass allocation **(A)** and phosphorus allocation **(B)** in *Solidago canadensis* forming mutualism with arbuscular mycorrhizal fungi (AMF). Available P, P was added as KH_2_PO_4_; Insoluble P, P was added as Ca_5_(PO_4_)_3_(OH); No P addition, no P was added. Error bars are the S.E. (*n* = 5). Different letters indicate a significant difference at *p* < 0.05.

Finally, we found that *S. canadensis* changed its resource allocation when it was colonized by *G. intraradices*. In insoluble P conditions, colonized plants allocated 47.5% more resources to above-ground growth while decreasing the biomass allocation to below-ground (Figure 6A and Table 2). Plants with AMF achieved a higher P content despite allocating fewer resources to below-ground growth and having decreased phosphatase activity (Figure 6B and Table 2). The effective resource allocation was higher in the No P addition treatment than in the Available P treatment (Figure 6B).

**TABLE 2:** Analyses of variance due to the effects of different phosphorus (P) treatments on the effective resource allocation of *Solidago canadensis*.

Measures	*F*2, 12	*p*-value
Below-ground dry mass	2.32	0.141
Above-ground dry mass	13.21	**0.001**
Phosphatase activity	5.19	**0.024**
Total phosphorus content	10.80	**0.002**

*Values of p < 0.05 are in bold.*

## Discussion

Our findings provide the first direct proof that AMF increase P uptake, change plant resource allocation strategy, and promote the growth of *S. canadensis* under conditions deficient in inorganic phosphorus. The efficient use of limited resources facilitated by mutualism with AMF contributes to the rapid growth of invasive weed*iS. canadensis* and may facilitate the invasion of new habitats.

### The Importance of Axenic Systems in Arbuscular Mycorrhizal Fungi Studies

The AMF colonization rate in our study (up to 83%) was substantially higher than in most previous studies, where they ranged from 21 to 73% (Li et al., 2006; Balzergue et al., 2011; Yuan et al., 2014; Hack et al., 2019). One reason for this could be that the axenic systems eliminate interference from other phosphorus solubilizing bacteria (Spagnoletti et al., 2017). Plants may need to invest more resources in maintaining a mutualistic relationship with AMF in the absence of these bacteria.

In non-axenic systems, the presence of other endophytes or rhizosphere bacteria has been found to promote both above- and below-ground growth (Calonne-Salmon et al., 2018; Zhan et al., 2018). In contrast, we found that AMF allow plants to reduce the amount of resources used to construct a root system and allocate more resources to above-ground structures in this study (Figure 4). This difference in results suggests that the presence of other microorganisms in non-axenic systems can obscure the true effects of AMF.

### Arbuscular Mycorrhizal Fungi Contribute to Phosphorus Uptake in Low Nutrient Conditions

Arbuscular mycorrhizal fungi can play significant roles in plant nutrient absorption, especially in nutrient-poor soil (Mikkelsen et al., 2008; Eissenstat et al., 2015). In our study, *S. canadensis* in the Insoluble P treatment accumulated more than twice as much P in the presence of AMF compared to their absence (Figure 5B). This finding is consistent with previous work by Li et al. (2006) and Yang et al. (2012), who found that over half of the P uptake by plants was due to AMF in soils with low P bioavailability. The mechanism underpinning this process is relatively well-understood. AMF secrete organic acids, phosphatases, and inorganic phosphorus transporters that contribute to the solubilization of insoluble P and the release of orthophosphate, which enhances P uptake and facilitates plant growth (Joner et al., 2000; Koide and Kabir, 2000; Bagyaraj et al., 2015). *S. canadensis* in a P-deficient environment secretes only half the amount of phosphatase with AMF colonization compared to no AMF colonization (Figure 5A). These findings were consistent with the idea that plants profit more from AMF whose hyphae would secrete phosphatases when insoluble P is available, allowing plants to decrease the resources allocated to phosphatase activity (Priyadharsini and Muthukumar, 2017). Evidence suggests that association with AMF leads to increase plant growth (Figures 3, 4, 6; Delavaux et al., 2017).

### Effects of Arbuscular Mycorrhizal Fungi in the Allocation Strategies in Invasive Plants

**FIGURE 7:**
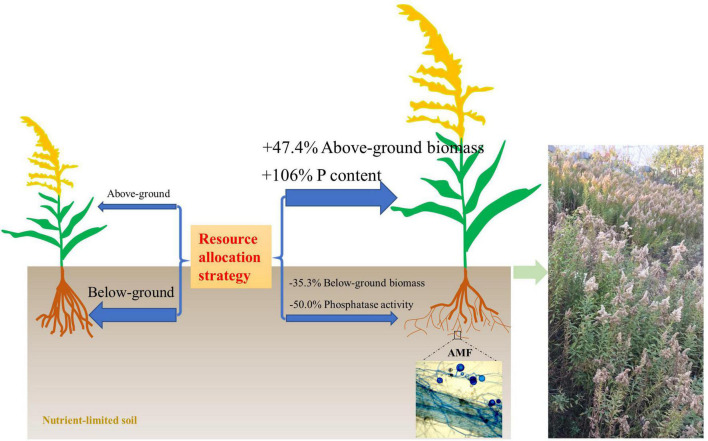
A model of the resource allocation strategy with/without arbuscular mycorrhizal fungi (AMF) on the growth of invasive weed *Solidago canadensis* in nutrient-limited soil.

There is still some debate as to whether invasive plants receive a greater benefit from mutualism with AMF than native species. Bunn et al. (2015) showed that native and invasive plants did not respond differently to AMF, but invasive plants had a higher level of AMF colonization when grown in competition with native plants. However, Menzel et al. (2017) showed that a mycorrhizal mutualism could promote the invasion success of neophyte plant species. The presence of arbuscular mycorrhiza has also been closely linked with plant invasions through facilitating nutrient cycling (Jo et al., 2018). Consistent with this, we found that AMF facilitate the growth of invasive plant *S. canadensis*. In nutrient-limited soil, *S. canadensis* allocates fewer resources to above-ground growth but more resource to below-ground growth for foraging more nutrients without AMF colonization. However, due to the presence of AMF, *S. canadensis* changes its resource allocation strategy and is able to allocate more resources to above-ground growth and facilitate phosphorus (P) uptake by the plant, allowing plants to have lower investment into below-ground biomass, and higher benefit/return for above-ground biomass (Figure 6, and schematic on the right in Figure 7). This is consistent with what Zhang et al. (2015) found, i.e., the colonization of AMF led to higher allocation to shoot biomass in rice. Shen et al. (2020) found that AMF increased P acquisition of the invasive *Eupatorium adenophorum*, and the P acquisition in above-ground was higher than in roots, which is consistent with our results.

In conclusion, we have shown that colonization by AMF is associated with changes in P uptake and increased growth in *S. canadensis*. With the contribution of AMF, the clonal plant *S. canadensis* is able to allocate more resources to above-ground growth, which might also affect its clonal performance. As a consequence, association with AMF likely contributes to *S. canadensis*’ success as an invasive clonal species, particularly in nutrient-limited habitats.

## Data Availability Statement

The original contributions presented in the study are included in the article/Supplementary Material, further inquiries can be directed to the corresponding author.

## Author Contributions

SQ and ZD designed the experiment, analyzed the data, and wrote the manuscript. SQ, JW, and LW performed the experiment. DS, DD, SE, SB, TT, and AM commented on the details of the manuscript drafts. All authors contributed critically to the drafts and gave final approval for publication.

## Conflict of Interest

The authors declare that the research was conducted in the absence of any commercial or financial relationships that could be construed as a potential conflict of interest.

## Publisher’s Note

All claims expressed in this article are solely those of the authors and do not necessarily represent those of their affiliated organizations, or those of the publisher, the editors and the reviewers. Any product that may be evaluated in this article, or claim that may be made by its manufacturer, is not guaranteed or endorsed by the publisher.
