# Supplementary material for: Arbuscular Mycorrhizal Fungi Contribute to Phosphorous Uptake and Allocation Strategies of Solidago canadensis in a Phosphorous-Deficient Environment
Source: Front Plant Sci. 2022 Mar 24;13:831654. doi: 10.3389/fpls.2022.831654 (PMC8987128; doi:10.3389/fpls.2022.831654)
Supplement: Supplementary file 1 [file Data_Sheet_1.docx]

Supplementary Material

## Supplementary Figures


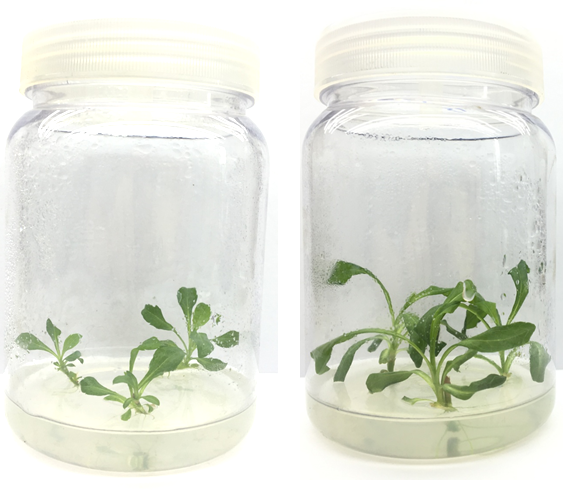


**Supplementary Figure 1.** Brief production process and propagation of *Solidago canadensis* aseptic seedlings. Sterilized fresh apical buds grow in Murashige and Skoog (MS) solid medium, when the clusters of axillary buds were ready, the shoots of proliferated seedling were cut and further cultured in MS solid medium till rooting to product complete aseptic seedling.


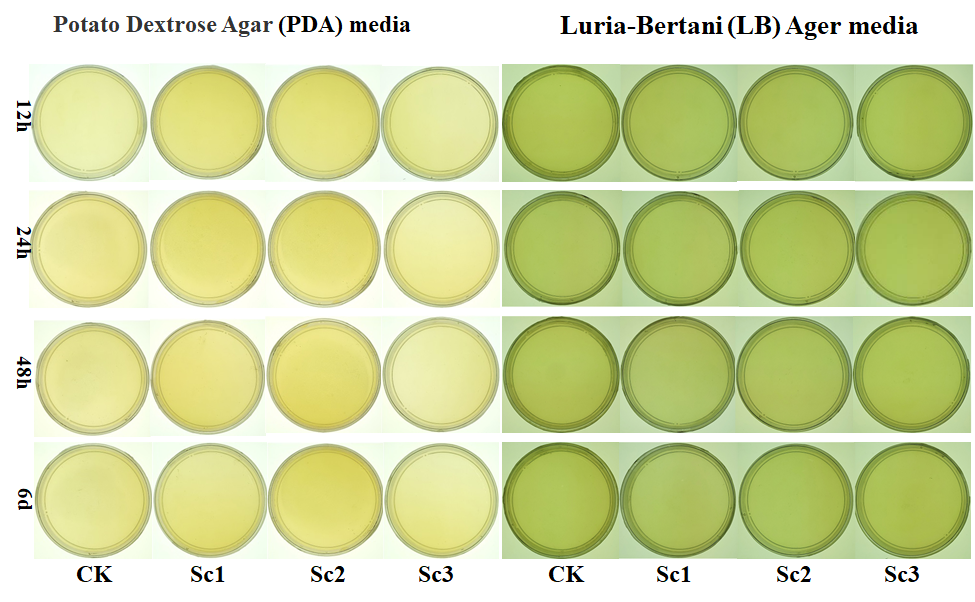


**Supplementary Figure 2.** Incubated plates of seedling grinding fluid on 12 h, 24 h, 48 h, and till 6 days showing no microorganisms inside seedlings. (Sc1-Sc3: 3 individual *Solidago canadensis* seedlings; CK: sterilized phosphate buffer saline, PBS; PDA ager media for the detection of fungi and LB ager media for the detection of bacteria)


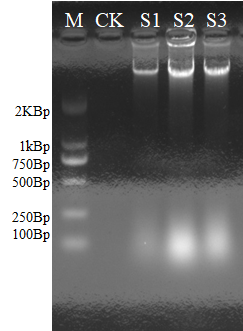


**Supplementary Figure 3.** Total DNA of 3 individual *Solidago canadensis* seedlings.

(M: DL2000 marker; CK: sterilized phosphate buffer saline (PBS); S1-S3: 3 individual *Solidago canadensis* seedlings)


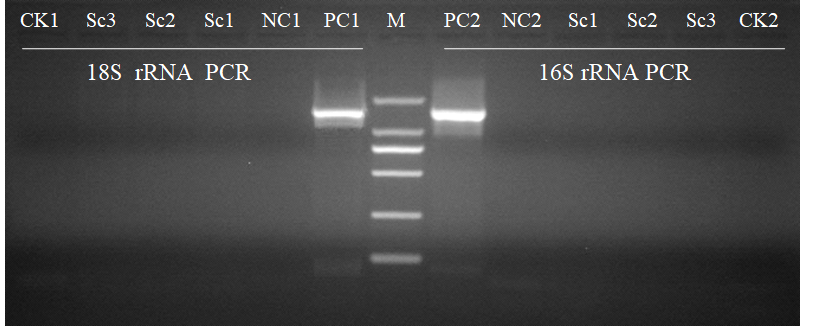


**Supplementary Figure 4.** The PCR analysis of 18S-rRNA and 16S-rRNA showing no microorganisms inside seedlings.

(M: DL2000 marker, PC1: 18S-rRNA PCR positive control- DNA template of *Rhizoctonia solani*; PC2: 16S-rRNA PCR positive control- DNA template of *Escherichia coli*; NC1 and NC2: PCR negative control -_dd_H_2_O; CK1 and CK2: sterilized phosphate buffer saline (PBS); Sc1-Sc3: 3 individual *Solidago canadensis* seedlings)


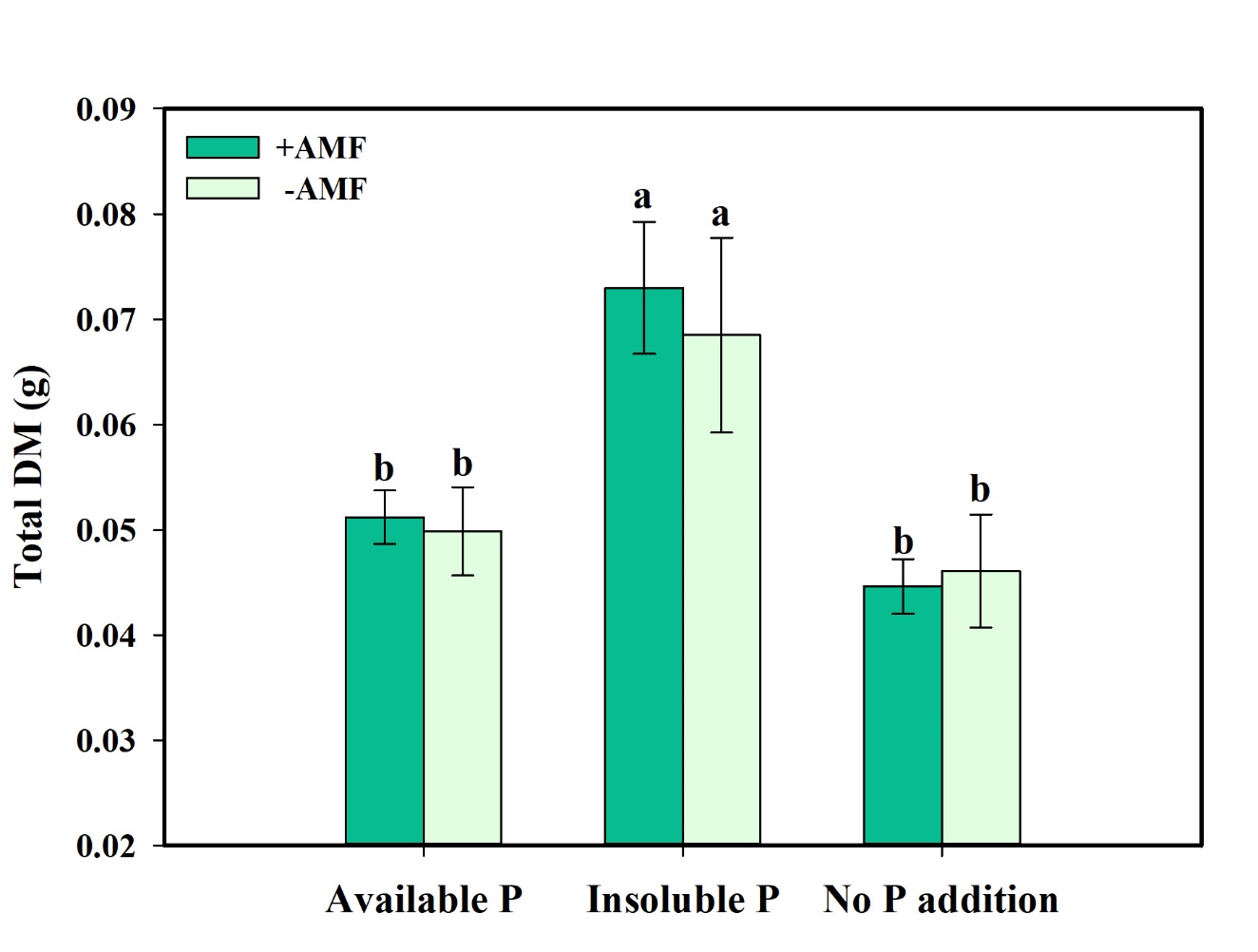


**Supplementary Figure 5.** Total dry mass of *Solidago canadensis* in different phosphorus (P) treatments. Available P, P was added as KH_2_PO_4_; Insoluble P, P was added as Ca_5_(PO_4_)_3_(OH); No P addition, no P was added. +AMF, with arbuscular mycorrhizal fungi (AMF) colonization; -AMF, without AMF colonization. Error bars are the S.E. (n = 5). Different letters indicate a significant difference at *p*< 0.05.
